# Coacervate whey protein improves inflammatory milieu in mice fed with high-fat diet

**DOI:** 10.1186/1743-7075-11-15

**Published:** 2014-03-28

**Authors:** Mayara Franzoi Moreno, Gabriel Inácio de Morais Honorato de Souza, Ana Claudia Losinskas Hachul, Bruno dos Santos, Marcos Hiromu Okuda, Nelson Inácio Pinto Neto, Valter Tadeu Boldarine, Elisa Esposito, Eliane Beraldi Ribeiro, Claudia Maria da Penha Oller do Nascimento, Aline de Piano Ganen, Lila Missae Oyama

**Affiliations:** 1 Departamento de Fisiologia, Disciplina de Fisiologia da Nutrição, Universidade Federal de São Paulo, São Paulo, SP, Brazil; 2 Instituto de Ciências e Tecnologia da Universidade Federal de São Paulo, São José dos Campos, SP, Brazil

**Keywords:** Mesenteric adipose tissue, Cytokines, IL-10

## Abstract

**Background:**

Functional foods with bioactive properties may help in treat obesity, as they can lead to a decreased risks of inflammatory diseases. The aim of this study was to investigate the effects of chitosan coacervate whey protein on the proinflammatory processes in mice fed with high-fat diet.

**Methods:**

Mice were divided into two groups receiving either a normolipidic or high-fat diet; the animals in each of the two diet groups were given a diet supplement of either coacervate (gavage, 36 mg protein/kg of body weight) or tap water for four weeks [groups: normolipidic diet plus water (C); normolipidic diet and coacervate (CC); high-fat diet and water (H); and high-fat diet and coacervate (HC)].

**Results:**

The high-fat diet promoted inflammation, possibly by decreased adiponectin/sum of adipose tissues ratio and increased phosphorylation of NF-κB p50. In HC we observed a positive correlation between IL-10 and TNF-α in mesenteric adipose tissue, retroperitoneal adipose tissue and liver tissue. We also observed a positive correlation between lipopolisaccharide with IL-10 in the liver tissue.

**Conclusions:**

High-fat diet treatment promoted metabolic alterations and inflammation, and chitosan coacervate whey protein modulated inflammatory milieu.

## Introduction

Obesity may be partly responsible for increased oxidative stress and consequent dysregulation of the expression of proinflammatory adipokines in different tissues or cells, which can trigger diseases related to metabolic syndrome [1]. However, the mechanisms by which fat accumulation directs the synthesis and secretion of adipokines needs to be further investigated.

Functional foods with bioactive properties may play an important role in combating obesity [2]. These properties may decrease the risk of diseases and enhance the antiinflammatory pathways [3,4]. However, formulation of functional foods with this potential is one of the challenges of the scientific community.

Faced with problems related to obesity and based on the literature, it is believed that whey protein (WP) as a functional food may be prophylactic against obesity and due its high nutritional value [2]. WP can act as antimicrobial agents, antihypertensive and as regulators of immune function, reducing body fat and may have a variety of related positive effects on human health [5-8]. Moreover, WP may function as an appetite suppressant [9] stimulating muscle protein synthesis and regulate homeostasis [7,9-12].

Chitosan coacervate WP is composed of by-products from the processing of shrimp, crab (chitosan) and cheese (WP), adding an environmental benefit to the product, as these by-products may be re-used and not disposed of in landfill sites or released into rivers by producers

The intake of high-fat diet rich in saturated fat is associated with the pathogenesis of obesity and metabolic diseases. Saturated fatty acids, in particular, favor a proinflammatory state leading to insulin resistance and are implicated in multiple inflammatory pathways, promoting lipotoxicity in several target organs by direct effects and indirectly by an alteration of gut microbiota associated with endotoxemia. Interactions between these pathways may perpetuate a feedback process that exacerbates an inflammatory state [13].

Fatty acids in excess have been show to induce hepatic insulin resistance and impair insulin clearance *in vitro* and *in vivo* in animal models [14,15] and humans [16]. Saturated fatty acids interferes with insulin signaling predominantly via intracellular kinases, which alter insulin receptor substrates, promoting deleterious effects in glucose and lipid metabolism [17,18].

Activation of intracellular kinases, such as the inhibitor of nuclear factor-κB kinase (IKK) and c-Jun N-terminal kinase (JNK), Toll-like receptor 4 (TLR-4), alter insulin receptor substrates and decrease insulin sensitivity. Additionally, activation of transcription factors may contribute to reduced glucose uptake by the expression of proinflammatory cytokines, such as TNF-α and IL-6, causing impairment in insulin receptor phosphorylation [17].

High-fat diet can lead to a significant change in the composition of dominant bacterial populations of gut microflora, including a decrease in the number of Bifidobacteria, Eubacterium rectal-Clostridium coccoides group and Bacteroides, favoring an increase in the gram-negative to gram-positive ratio. The change in bacterial populations is associated with significant increases in plasma lipopolysaccharide (LPS) levels, which are recognized in the circulation (endotoxemia) by toll-like receptors in the cell membranes and activate specific kinases, which lead to insulin resistance. These pathways also activate NF-ĸB, which results in the expression of inflammatory genes. Similar to LPS, saturated fatty acids are recognized by membrane receptors that trigger pro-inflammatory signaling pathways [19,20]. The anti-inflammatory effect of adiponectin has been well documented [21,22]. However the effects of chitosan coacervate whey protein on metabolic inflammatory pathways are not well elucidated.

Considering these complex inflammatory mechanisms, continual investigation of functional compounds capable of eliciting anti-inflammatory reactions and inhibiting the inflammatory cascade signalization will aide in understanding the causes of obesity and obesity-related diseases.

In addition, reviewing the literature, few scientific studies have been conducted to date that justifies the realization of this experimental study, with the purpose of elucidating scientific parameters for preventing and for therapeutics in obesity.

For this, it is necessary to understand the possible changes involved in supplementation of chitosan coacervate WP and proinflammatory processes triggered by high-fat diet.

## Materials and methods

### Animals and treatment

Fifty male Swiss mice aged 51 days were acquired from CEDEME (Centro de Desenvolvimento de Modelos Experimentais da Universidade de São Paulo) and kept under controlled conditions of light (12 h light–dark cycle with lights on at 6 am) and temperature (24 ± 1°C), in collective cages (5 mice per cage, four cages) for a week of acclimation. After this period, all mice were given water and food (two cages given normolipidic (control) diet, and two high-fat diet) *ad libitum* for five weeks (cycle I), followed by four weeks (cycle II) with coacervate (36 mg/kg/day) or vehicle (tap water) treatment by gavage [(C) control diet plus tap water; (CC) control diet plus coacervate; (H) high-fat diet plus tap water; and (HC) high-fat diet plus coacervate]. All diets were prepared according to the recommendations of the American Institute of Nutrition [23] (Table 1). To manufacture the coacervate were used: Serum sweet milk fat free lyophilized provided by the company Libra®, chitosan with average molecular weight, viscosity 200,000 cps, with a degree of deacetylation of 85-95% (Sigma-Aldrich, Inc. St. Louis, MO, USA), citric acid (Lot: 40053 Fmaia industry and Trade Ltd.) and sodium hydroxide (Lot: 109062 Merk CHEMICAL SA). The body weight gain was monitored twice a week. The experimental research committee of the Universidade Federal de São Paulo approved all procedures for the care of the animals used in this study (CEP 114254).

**Table 1:** Macronutrients and micronutrients composition of the normolipidic and high-fat diet AIN-93 M (g/kg diet)

**Nutrients**	**Normolipidic diet (g/kg)**	**High-fat diet (g/kg)**
Carbohydrates (g)	720.7	408.7
Carbohydrates (kcal)	76%	30.5%
Protein (g)	140	140
Protein (kcal)	14.5%	10.5%
Lipids (g)	40*	352^†^
Lipids (kcal)	9.5%	59%
Fiber (g)	50	50
Vitamin mix (g)	10	10
Mineral mix (g)	35	35
L-Cysteine (g)	1.8	1.8
Choline bitartrate (g)	2.5	2.5
Tert-butylhydroquinone (mg)	8	8
Energy value	3.8 kcal/g	5.36 kcal/g

*40 g soybean oil; ^†^312 g lard plus 40 g soybean oil.

### Oral Glucose Tolerance Test (OGTT)

After 12 h overnight fast, blood was collected from the tail vein to assess basal glucose concentration. Then, a glucose (Merck®) solution (1.4 g/kg) was administrated by gavage. Blood samples were collected after 15, 30, 43, 60 and 120 minutes to measure glucose concentration using a glucose analyzer (AccuCheck Roche®).

### Experimental procedures

At the end of the experimental period, animals were fasted for 12 h overnight prior to being sacrificed by decapitation. Trunk blood was collected and immediately centrifuged (1125 g/15 min at 4°C). Serum was separated and stored at −80°C for later biochemical and hormonal determination. The adipose tissue depots: retroperitoneal (RET), mesenteric (MES) and epididymal (EPI), and liver were dissected, weighed, immediately frozen in liquid nitrogen and stored at −80°C.

### Biochemical and hormonal serum analyses

Glucose, TC, TG and HDL serum concentrations were measured by an enzymatic colorimetric method using commercial kits (Labtest®, Brazil). Insulin and adiponectin concentrations were quantified using specific enzyme-linked immunosorbent assay (ELISA) kits (Milipore and R&D Systems®). The LPS level was determined using commercial kits (Lonza®).

### Protein analysis by Western Blotting

After euthanasia, MES was rapidly removed, homogenized in 1.0 mL extraction buffer (100 mM Trizma, 1% SDS, 100 mM sodium pyrophosphate, 100 mM sodium fluoride, 10 mM EDTA and 10 mM sodium orthovanadate). The extracts were then centrifuged at 20,817 g at 4°C for 40 min to separate the protein extract. Protein determination was performed by the Bradford dye method using the Bio-Rad reagent (Bio-Rad Laboratories®, Hercules, CA, USA). The proteins were treated with Laemmli sample buffer containing dithiothreitol and heated at 100°C for 5 min before loading onto 8 or 10% SDS-PAGE in a Bio-Rad miniature slab gel apparatus. Electrotransfer of proteins from the gel to the nitrocellulose membrane was performed for 1 h at 120 V (constant) in a Bio-Rad semi-dry transfer apparatus. Nonspecific protein binding to the nitrocellulose was reduced by pre-incubation for 2 h at 22°C in blocking buffer (1% bovine serum albumin, 10 mM Tris, 150 mM NaCl and 0.02% Tween 20). The membranes were incubated overnight at 4°C with antibodies against p-NF-kBp50 (sc-101744, diluted 1:1000), p-NF-kBp65 (sc-71675, 1:1000), p-IκB (sc-847, 1:1000), TLR4 (sc-10741, 1:1000), SOD-1(271014, 1:1000), GPX-3 (sc-50496, 1:1000) and α-Tubulin (sc-58667, 1:1000) obtained from Santa Cruz Biotechnology® (Santa Cruz, CA, USA), diluted in blocking buffer combined with 1% bovine serum albumin (BSA) and then washed 3 × 10 min in blocking buffer without BSA. The blots were subsequently incubated with a peroxidase-conjugated secondary antibody for 1 h at 22°C in blocking buffer and processed for enhanced chemiluminescence using (Amersham ECL) to visualize the immunoreactive bands using Uvitec Alliance 4.7 Cambridge®. Band intensities were quantified by optical densitometry (Scion Image-Release Beta 3b, NIH, USA) of the developed autoradiographs.

### IL-6, IL-10 and TNF-α protein level determined by ELISA

Following decapitation, the adipose tissue depots RET (0.3 g), MES (0.3 g), EPI (0.3 g), and liver (0.1 g) were homogenized in 800 μL of chilled extraction buffer (100 mM Trizma Base pH 7.5; 10 mM EDTA; 100 mM NaF; 10 mM Na_4_P_2_O_7_; 10 mM Na_3_VO_4_; 2 mM PMSF; 0.1 mg/mL aprotinin). After homogenization, 80 μl of 10% Triton X-100 were added to the samples, which were left in ice for 30 minutes and then centrifuged (20,817 g, 40 minutes, 4°C). The supernatant was saved, and protein concentration was determined using the Bradford assay [24] (Bio-Rad, Hercules, California) with bovine serum albumin as a reference. Quantitative assessment of TNF-α, IL-6 and IL-10 proteins was carried out by ELISA (DuoSet ELISA, R&D Systems, Minneapolis, MN, USA) following the recommendations of the manufacturer. All samples were run in duplicate, and the mean value was reported.

### Statistical analysis

All results are presented as mean ± standard error of the mean (SEM). Statistical significances were assessed using two-way analysis of variance (ANOVA) followed by Tukey’s *post hoc* analysis to identify significant differences among the groups. The *Pearson’s* correlation was used to assess the associations between the analyzed variables. Differences were considered significant for (p ≤ 0.05) with the StatsDirect® software.

## Results

### Body weight, delta body weight and Oral Glucose Tolerance Test (OGTT)

**Figure 1:**
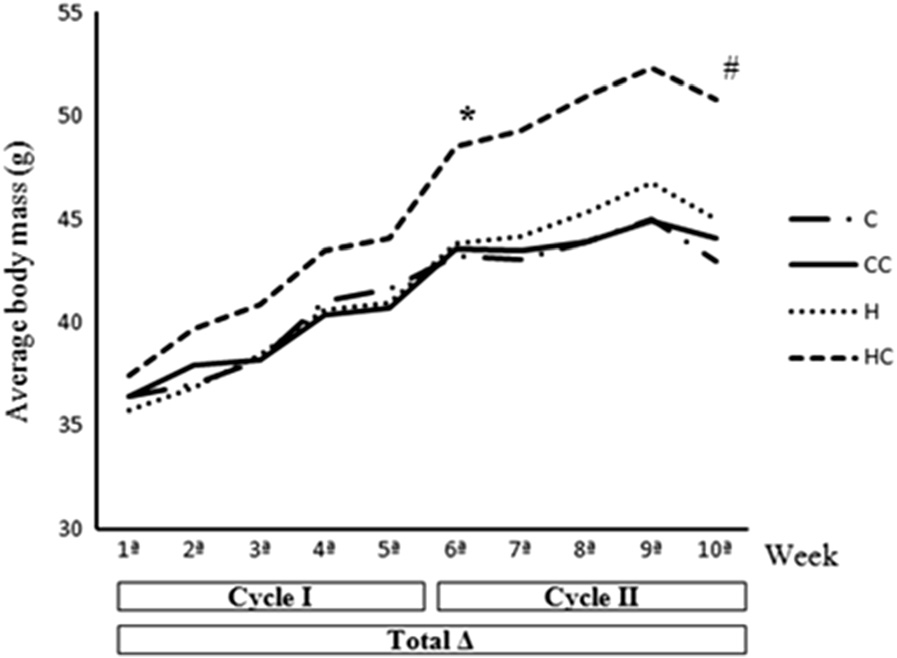
**Average body mass (g) per week in the experimental groups treated with high-fat or control diet (cycle I); and high-fat diet or control associated with gavage of water or coacervate (cycle II); (n = 9-14).** *different from C (p = 0.04); ^†^different from C, CC and H (p < 0.02).

Significant differences were observed in the delta (g/100 g body weight) of cycle I, between groups C and HC (6.03 ± 0.90 vs 10.53 ± 1.46, respectively; p = 0.04). In cycle II with gavage of coacervate we did not observe significant differences between the groups. However, when we consider the whole period of experimentation (total delta) the HC group (16.70 ± 3.09) is statistically different when compared to the other three groups HC *vs* C (6.61 ± 0.44; p = 0.001), HC *vs* CC (7.53 ± 1.63; p = 0.01), HC *vs* H (9.53 ± 1.63; p = 0.02) (Figure 1).

The first OGTT was performed at the end of cycle I, but there were no significant differences in its area under the curve (AUC). The same was observed for the second OGTT at the end of cycle II, indicating no effect of coacervate ingestion on oral glucose tolerance in mice (data not showend).

### Relative weight of tissues (g/100 g body weight)

The liver relative weight in the HC group (3.24 ± 0.07) was significantly lower than the C group (3.77 ± 0.08) (p = 0.01). There were no differences in MES relative weight among groups. The relative EPI was higher in groups H (4.88 ± 0.32) (p = 0.04) and HC (4.64 ± 0.18) (p = 0.03) versus control (3.28 ± 0.23). The RET relative weight was higher in HC (1.32 ± 0.12) (p = 0.04) *vs* control (1.0 ± 0.41). The sum of adipose tissues (SAT) relative to weight in HC (8.84 ± 0.53) was higher (p = 0.007) *vs* control (5.76 ± 0.41) (data not showed).

### Serum cholesterol, HDL, glucose, LPS, triacylglycerol (TG), adiponectin and insulin concentrations

Serum cholesterol, HDL, TC, LPS, glucose and insulin did not differ among the groups, except TG in the hyperlipidic were lower in concentration than the control group (H; p = 0.008) and (HC; p = 0.01).

The concentrations of adiponectin are based on the amount of fat mass, remember this relationship is inversely proportional, higher values of SAT are shown correlated with lower values of adiponectin. The ratio of adiponectin/SAT was calculated to remove this effect on the data. We present the values of adiponectin serum levels and the adiponectin concentrations on the basis of SAT and as percentage of the control amount. There were a significant decrease in serum levels of adiponectin in H (p = 0.02) compared with CC. The adiponectin/SAT ratio was higher in C than in H (p < 0.003) and HC (p = 0.01) (Table 2).

**Table 2:** Glucose (mg/dL), TC (mg/dL), HDL (mg/dL), LPS (EU/mL), TG (mg/dL), adiponectin (ng/mL), adiponectin/sum of adipose tissue (SAT) and insulin (ng/mL) concentrations in the experimental groups

	**C**	**CC**	**H**	**HC**
**Glucose (mg/dL)**	121.82 ± 11	123.77 ± 14.24	134.58 ± 10.8	132.82 ± 11.73
**TC (mg/dL)**	147.69 ± 7.97	135.79 ± 13.30	152.74 ± 7.73	151.37 ± 7.57
**HDL (mg/dL)**	64.77 ± 7.97	87.9 ± 11.25	78.51 ± 4.22	75.34 ± 4.97
**LPS (EU/mL)**	4.94 ± 1.85	1.90 ± 0.29	2.45 ± 0.41	2.39 ± 0.26
**TG (mg/dL)**	173.51 ± 10,53	160,15 ± 8,34	130.68 ± 0.54^†^	136.19 ± 5.17^†^
**Adiponectin (ng/mL)**	4.01 ± 0.15	4.11 ± 0.11	3.22 ± 0.22*	3.64 ± 0.22
**Adiponectin/SAT (%)**	101.9 ± 7.26	87 ± 8.83	54.66 ± 4.23^†^	59.95 ± 7.10^†^
**Insulin (ng/mL)**	0.66 ± 0.16	0.95 ± 0.22	0.75 ± 0.09	0.98 ± 0.09

Data are mean ± SEM (n = 8); ^†^different from C (p ≤ 0,01); *different from CC (p = 0.02).

### Tissues cytokines content

We found that IL-6 content in RET was increased in CC as compared to the HC (p = 0.01) group. In the MES, the IL-6 content in CC (p = 0.03) was increased in relation to the HC group.

The IL-10/TNF-α ratio was lower, in MES of C compared to H (p = 0.0009). The IL-10/TNF-α ratio in liver tissue was increased in C compared to H (p = 0.02), and CC was lower compared to the HC (p = 0.008).

In all tissues, TNF-α content showed no significant differences among the C, CC, H and HC groups (Table 3).

**Table 3:** Concentrations (pg/mg protein) TNF-α. IL-10 and IL-6 in different adipose tissue and liver in the experimental groups

		**MES**	**EPI**	**RET**	**Liver**
**TNF-α**	**C**	30.97 ± 6.59	34.87 ± 4.45	53.64 ± 9.16	2.06 ± 0.11
	**CC**	51.21 ± 12	47.06 ± 6.39	52.18 ± 7.69	2.18 ± 0.2
	**H**	33.58 ± 8.02	30.43 ± 2.45	56 ± 9.64	2.87 ± 0.25
	**HC**	29.58 ± 5.2	30.71 ± 6.84	35.15 ± 5.94	2.33 ± 0.26
**IL-10**	**C**	15.43 ± 2.75	17.08 ± 1.94	30.69 ± 2.96	4.87 ± 0.38
	**CC**	28.21 ± 7.23	30.66 ± 2.85	32.54 ± 3.48	4.16 ± 0.42
	**H**	32.39 ± 6.81	15.47 ± 1.37	26.17 ± 3.68	4.72 ± 0.63
	**HC**	23.75 ± 5.24	18.18 ± 4.95	21.6 ± 2.15	3.23 ± 0.30
**IL-6**	**C**	55.05 ± 6.30	41.81 ± 4.41	92.43 ± 13.83	14.86 ± 1.93
	**CC**	74.67 ± 11.89	62.52 ± 5.67	114.48 ± 10.20	12.77 ± 0.48
	**H**	57.93 ± 11.56	35.27 ± 3.18	69.66 ± 14.63^†^	16.47 ± 1.49
	**HC**	39.02 ± 6.73*	36.18 ± 7.41	61.01 ± 8.67*	12.98 ± 1.37
**IL-10/TNF-α**	**C**	0.38 ± 0.08	0.52 ± 0.05	0.58 ± 0.06	2.26 ± 0.17
	**CC**	0.53 ± 0.09	0.70 ± 0.06	0.59 ± 0.04	2.05 ± 0.27
	**H**	1.19 ± 0.18^†^	0.57 ± 0.11	0.49 ± 0.03	1.64 ± 0.14^†^
	**HC**	0.81 ± 0.16	0.49 ± 0.07	0.69 ± 0.10	1.50 ± 0.1

Data are mean ± SEM (n = 8); ^†^different from C (p ≤ 0.05); *different from CC (p ≤ 0.04).

### Western blot

**Figure 2:**
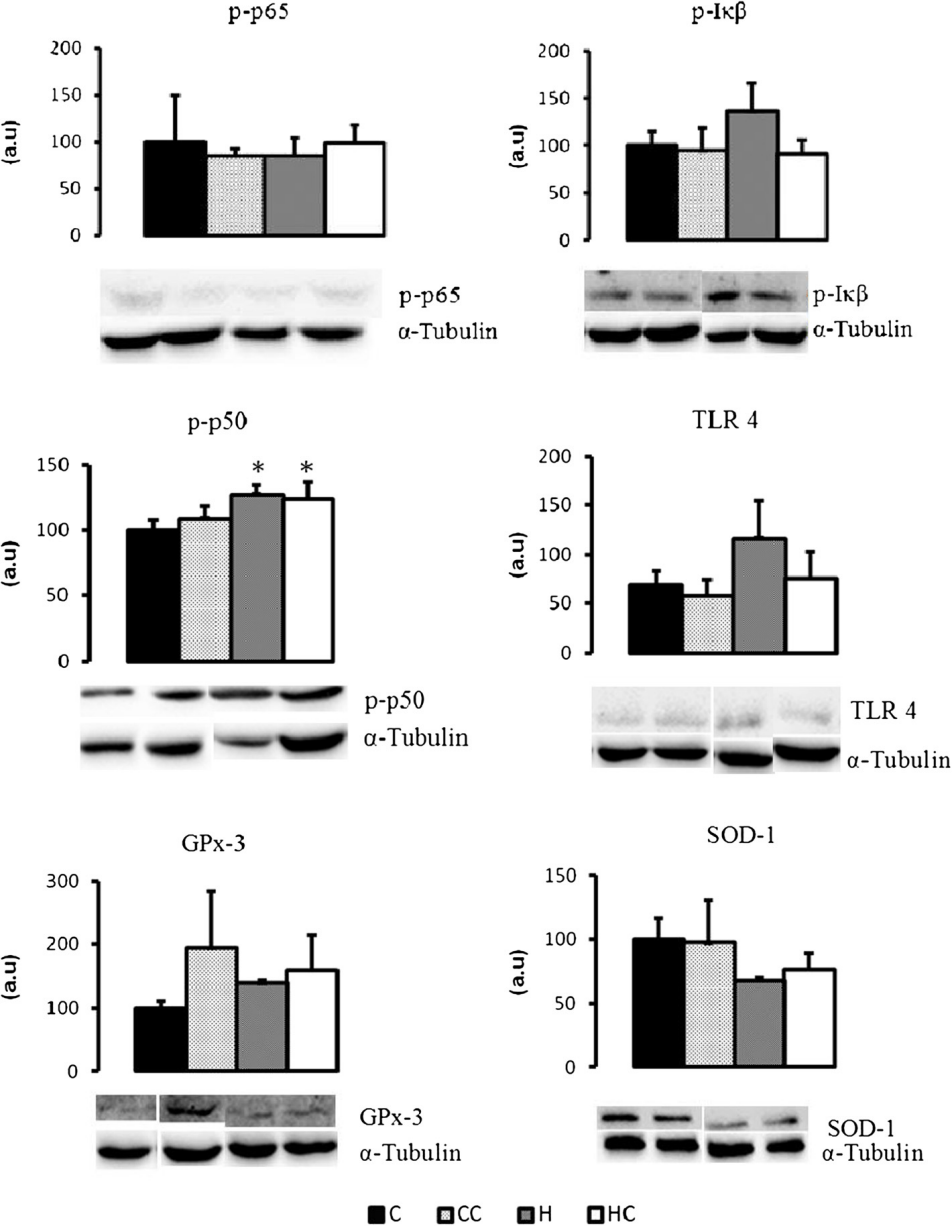
**Western blot analysis of p-pNF-kBp65, p-NF-kBp50, p-IκB, TLR4, GPx-3, SOD-1 protein in MES adipose tissue in different groups.** Data are mean ± SEM (n = 4–9); *different from C (p ≤ 0.01).

Following the analysis of tissues cytokines content, there was marked an effect of the treatment in the mesenteric adipose tissue deposits. Then, we studied the NF-kB complex in this tissue. Phosphorylation of NF-kB p50 in MES was increased in the groups H (p = 0.01) and HC (p = 0.003) compared to C group, as verified by Western Blot (Figure 2).

### Correlation between cytokines and LPS

**Figure 3:**
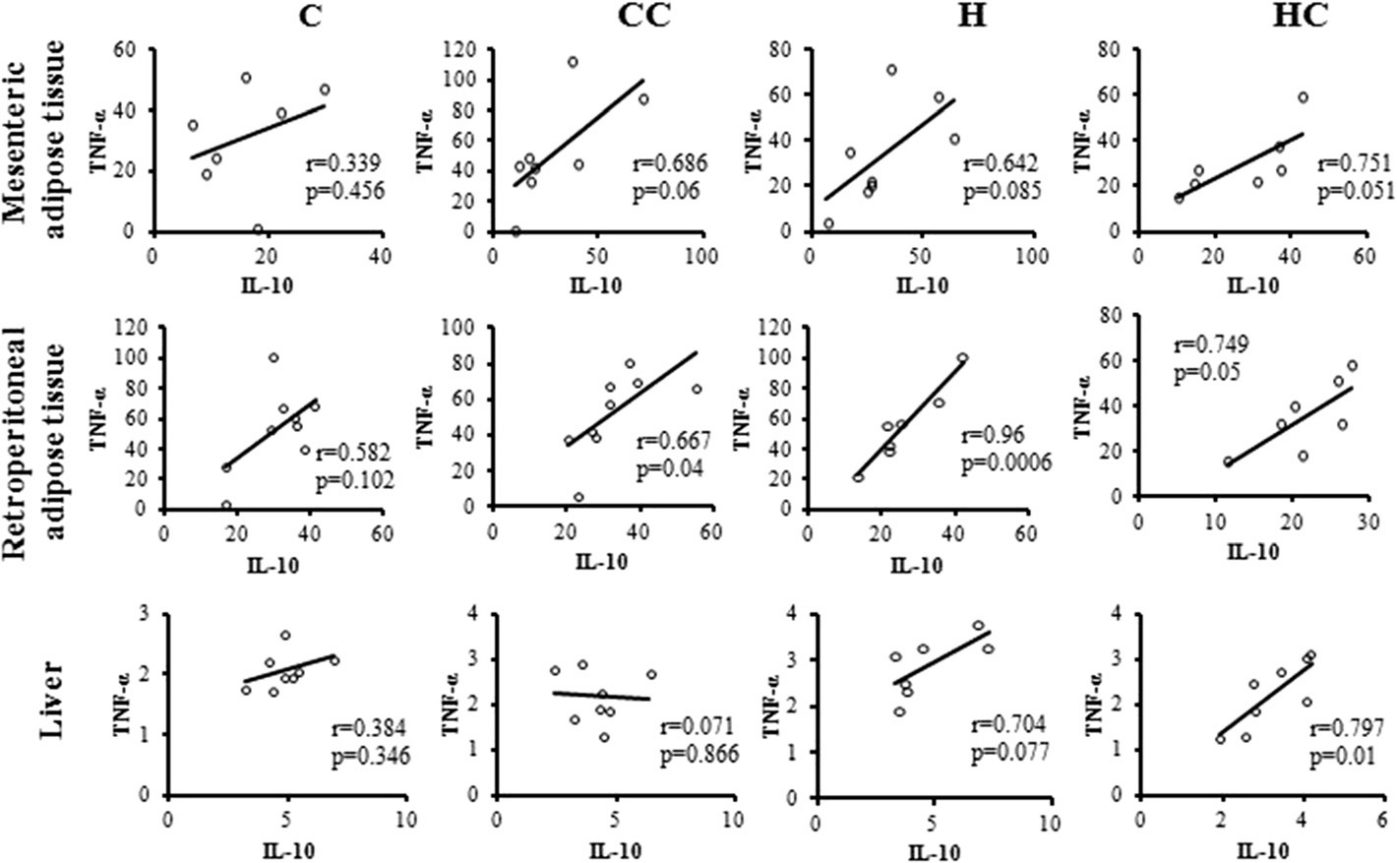
**Positive correlation between IL-10 and TNF-α in liver, mesenteric adipose tissue, and retroperitoneal adipose tissue.** Rows represent tissue, and columns groups (n = 7-9).

We found positive correlations between IL-10 and TNF-α in the HC group in adipose tissues RET (p = 0.05) (r) = 0.749, MES (p = 0.05) (r) = 0.751 and in liver tissue (p = 0.01) (r) = 0.797 (Figure 3). A positive correlation was also observed in H (RET tissue).

**Figure 4:**
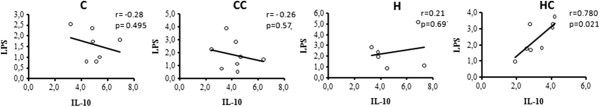
Correlation between LPS and IL-10 in liver (n = 6-8).

A positive association between LPS with IL-10 in the liver tissue in the HC group (Figure 4) was found when using Person’s correlation.

## Discussion

The intake of high-fat diet rich in saturated fat is associated with the pathogenesis of obesity and metabolic diseases. Several methods are used to minimize the effects of excessive body fat deposition, including foods rich in bioactive compounds. Results from previous studies have demonstrated the anti-obesity properties of compounds, such as whey peptides [2,12,25].

Our results suggest that treatment of *Swiss* mice with a high-fat diet for 9 weeks did not increase weight gain, findings which support the results of previous work [26,27], or induce significant changes in OGTT. Although the high-fat treatment did not induce changes in glucose homeostasis and weight, it was effective in triggering inflammatory processes as was seen in the decrease adiponectin/SAT ratio and the increased phosphorylation of the p50 subunit from the NF-κB complex in MES adipose tissue.

The lipopolysaccharides (LPS) and saturated fatty acids act on receptors of family *Toll Like Receptor* (TLR), in particular TLR4, activating the track of NF-κB, which favors the gene expression of pro-inflammatory adipokines [28,29]. Signal transmission mediated by connection between LPS and TLR4 constitutes a highly complex and varied phenomenon, mediated through reactions involving phosphorylation and ubiquitination of target proteins. Specifically involved is MyD88 protein activation of the complex IRAK (IL-1 receptor associated Kinase) -TRAF 6 (TNF receptor-associated factor), the latter belongs to the ubiquitin ligases class (E3 ligases) and appearsto be essential for the NF-κB uncoupling, its inhibitory protein (Iκ-B). NF-κB, once released, migrates to the nucleus by binding to DNA, starting the genic amplification of proteins related to inflammation [30].

TLR-4 activation by FFA or lipopolysaccharides (LPS) in adipocytes may be involved in the development of IR in obesity and DM2 [31,32].

Adipose tissue serves as an energy reserves in addition to functioning as an endocrine organ which secretes proteins and cytokines [33,34]. For example adiponectin, an adipokine secreted almost exclusively by adipose tissue increases insulin sensitivity and has anti-atherogenic effect by reducing the inflammatory process, and is usually found in lower amount in obese individuals [35,36].

In this experimental study we attempted to induce metabolic changes with the administration of a high-fat diet to mice for five weeks and subsequently treated the animals with chitosan coacervate WP for four more weeks concurrently with the diet. It was expected that the coacervate reduced body weight, similarly to other studies of WP, but our findings for coacervate WB were not consistent with the literature. The HC group animals showed an increase in body weight gain compared to the other experimental groups and SAT increased compared with group C. We hypothesize that the body mass gain of the HC animals was not due solely to fat depots, as was evident once differences in fatty tissue between the H and HC groups were not observed. Probably the body mass gain could be related to the increase of body fat free mass.

Analyzing the serum lipid profile, we found that H and HC groups showed lower concentrations of triacylglycerol than group C. This result can be explained by differences in the carbohydrate rate offers in the control and high-fat diet, where the high fat diet groups received a minor CHO rate. The high-fat diet possibly increased lipoprotein lipase activity and consequently stimulated the uptake of fatty acids by adipose tissue and reducing the hepatic lipogenesis, resulting in a decrease in serum TG [37,38].

The IL-10 is a pleiotropic cytokine that controls inflammatory processes by eliminating the pro-inflammatory cytokines production such as IL-1, IL-6, IL-8 and TNF-α is produced mainly by monocytes, macrophages, lymphocytes, mast cells and mature adipocytes [39]. The IL-10/TNF-α ratio has been considered an important indicator of inflammatory status as low values are often associated with increased morbidity and mortality risk [40,41].

Interestingly, we observed an increase of the IL-10/TNF-α ratio in MES of group HC despite the expectation of finding a reduced ratio in this group. We believe that because of the short period of the treatment the animals may still be attempting to reverse their pro inflammatory state, which increased IL-10/TNF-α ratio leading to an anti-inflammatory state. With increased length of treatment we may have found a reduced ratio of IL-10/TNF-α. According the results of this study coacervate may have protected the mice from a pro inflammatory state triggered by the treatment diet, since this ratio was equal to the control, however, a study involving a longer treatment period may be required to discern this possible effect.

Differences between mesenteric adipose tissue and other fatty tissues responses can be explained by the difference in inflammatory marker secretory capacity and in the number of resident macrophages in both depots. Cytokine production of macrophages is higher in MES, and these cells are important to adipose tissue maintenance being highly responsive to inflammatory effects [42].

Multiple intracellular pathways are involved in the secretion of newly synthesized IL-10 from macrophages following TLR4 activation with LPS, as well, trafficking pathways for IL-6 and TNF-α in macrophages can simultaneously produce pro-inflammatory cytokines [43]. Our results support this pathway due to the finding of a positive association between TNF-α, LPS with IL-10 in the liver tissue in the HC group. This result reinforces the view that the increase in the IL-10 concomitantly to LPS and TNF-α concentration is an important mechanism in reversing the inflammatory process.

The treatment time and the total body composition analysis can be considered as limitations of our study. A longer treatment time may lead to an increase an increase in body weight and alter the OGTT. Additionally, the fat free body mass measurement can explain the high body weight gain in the HC group. We showed the potential positive effects of coacervate whey protein supplementation on the metabolic profile and inflammatory pathways in mice fed with high-fat diet. We also provide evidence of the association between IL-10 and LPS in hepatic tissue when treated with coacervate. In future investigations os the effects of coacervate whey protein we suggest the evaluation of antioxidant enzymes activity, which may additionally illustrate the inflammatory cascade of TLR-4 activated by LPS, reflecting the endotoxemia caused by high-fat diet administration.

The inflammatory process is a complex reaction, in which the adipokines, cytokines, hormones and thousands of molecules appear to interact and play multiple roles on several metabolic pathways. In order to further validate the data previously presented. In addition, an analysis of correlation between IL-10 and TNF-α in MES adipose tissue, RET and liver of animals in the HC group, suggest an increase in the production of IL-10, could be useful in further elucidating the role of coacervate in reversing the inflammatory effets triggered by a high-fat diet.

## Abbreviations

WP: Whey protein; EPI: Epididymal; RET: Retroperitoneal; MES: Mesenteric; TC: Total cholesterol; HDL: HDL-cholesterol; IL-6 and IL-10: Interleukins 6 and 10; TNF-α: Tumor necrosis factor α; LPS: Lipopolisaccharide; TLR-4: Toll like receptor 4; OGTT: Oral glucose tolerance test.

## Competing interests

The authors declare that they have no competing interests.

## Authors’ contributions

All authors read and approved the final manuscript.
